# Investigation of Filamentous Fungi Producing Safe, Functional Water-Soluble Pigments

**DOI:** 10.1080/12298093.2018.1513114

**Published:** 2018-09-27

**Authors:** Young Mok Heo, Kyeongwon Kim, Sun Lul Kwon, Joorim Na, Hanbyul Lee, Seokyoon Jang, Chul Hwan Kim, Jinho Jung, Jae-Jin Kim

**Affiliations:** Division of Environmental Science & Ecological Engineering, College of Life Sciences & Biotechnology, Korea University, Seoul, Republic of Korea

**Keywords:** Acute toxicity, antioxidant, colorimetry, fungal pigment

## Abstract

The production of water-soluble pigments by fungal strains indigenous to South Korea was investigated to find those that are highly productive in submerged culture. Among 113 candidates, 34 strains that colored the inoculated potato dextrose agar medium were selected. They were cultured in potato dextrose broth and extracted with ethanol. The productivity, functionality (radical-scavenging activities), and color information (CIELAB values) of the pigment extracts were measured. Five species produced intense yellowish pigments, and two produced intense reddish pigments that ranked the highest in terms of absorbance units produced per day. The pigment extracts of *Penicillium miczynskii*, *Sanghuangporus baumii*, *Trichoderma* sp. 1, and *Trichoderma afroharzianum* exhibited high radical-scavenging activity. However, the *S. baumii* extract showed moderate toxicity in the acute toxicity test, which limits the industrial application of this pigment. In conclusion, *P. miczynskii* KUC1721, *Trichoderma* sp. 1 KUC1716, and *T. afroharzianum* KUC21213 were the best fungal candidates to be industrial producers of safe, functional water-soluble pigments.

## 1. Introduction

Pigments are colorants that have been used as additives to enhance the quality of products by intensifying their original color and dyeing colorless products [1]. The main sources of pigments, historically, were insects and plants until the demand for colorants drastically increased with the industrial revolution [2]. Because the production of natural pigments could not meet the market demand, synthetic colorants were developed and achieved market dominance. However, some synthetic colorants contained potential colon carcinogens and people have begun to worry about the health effects of synthetic colorants, especially in food, clothes, cosmetics, and pharmaceuticals [3–6]. This encouraged interest in developing non-toxic colorants and broadening the sources of natural pigments. The natural colorant market started to grow and is expected to reach 2.3 billion dollars in 2019 [7]. The successful marketing of algal pigments proved that the willingness to pay for natural healthy colorants has increased [8].

On the other hand, the market price of natural colorants is high due to their low production levels [9]. Several other problems, such as a lack of stability or pH-dependency, also exist [2]. To overcome these obstacles, researchers have suggested that microorganisms, including bacteria or fungi, should be developed and utilized as alternative sources of conventional natural pigments [2].

Filamentous fungi are well known for producing various secondary metabolites that are useful to humans [10]. As examples of such metabolites, fungal pigments have many advantages as natural colorants; they are biodegradable (eco-friendly), have various colors, exhibit diverse chemical profiles, are mostly non-toxic, are generated from cheap raw materials, and are easily produced at high levels [11]. In addition, many fungal pigments exhibit useful physiological activities, including antimicrobial, antimutagenic, antioxidant, herbicidal, anticancer, and antiobesity activities [12–14]. The functionality of fungal pigments makes their value even higher. In particular, pigments with high antioxidant activity are very useful and easily applied in the food and cosmetic industries.

However, like synthetic colorants, fungal pigments are not free from safety concerns. Pigments from *Monascus* species have been widely used in China for a long time and are probably the best- known fungal pigments globally. However, broadening their market to Europe or USA has been difficult due to safety concerns about their citrinin content. Citrinin is a yellow pigment that is a potential carcinogen, causing nephrotoxic, hepatotoxic, and cytotoxic effects. Both *Monascus ruber* and *M. purpureus,* the two major species in the genus, have been reported to produce citrinin, and its production is difficult to prevent because its biosynthetic pathway is shared with pigment synthesis [15,16]. Toxin-producing fungi have a major disadvantage in that the presence of the toxins strongly limits the application range of their pigment products, such as use in the food and beverage industries. In addition, since toxic products must be diluted or detoxified prior to their discharge into rivers, the cost of wastewater treatment increases proportionally to toxicity, leading to higher unit costs. Fungal strains that produce pigments, but not toxins, should be found and developed to avoid these problems.

Some fungal pigments have already been introduced to the market and are now being used: β-Carotene and lycopene are produced from *Blakeslea trispora*, lactoflavin is produced by *Ashbya gossypii*, and a commercial colorant, “Arpink Red,” is produced by *Penicillium oxalicum* [7]. The yield of β-carotene from *B. trispora* was reported to be at least 9 grams per liter of culture medium [17]. These successes prove that fungal pigments can be approved and commercialized with sufficient competitiveness.

Water-soluble pigments are more suitable for industrial production because they are easy to mass-produce using bioreactors and are easily extracted without costly and hazardous organic solvents. For these reasons, finding fungal strains that can produce large amounts of water-soluble pigments in submerged culture is important. In this study, we explored indigenous fungi in South Korea to find fungal strains that produce water-soluble pigments in submerged culture, and the productivity, color information, functionality, and toxicity of their extracted pigments were examined.

## 2. Materials and methods

### 2.1. Fungal candidates

All the fungal strains used in this study were obtained from the Korea University Culture (KUC) collection. Fungal species belonging to the same genera of fungi reported to produce pigments were selected as potential pigment-producing fungi. Several other strains of the KUC collection were also selected based on random observation of fungal cultures. Species reported to produce toxins were then excluded.

### 2.2. Molecular identification

Fungal DNA was extracted using an AccuPrep Genomic DNA Extraction Kit (Bioneer, Seoul, Korea), and the nuclear ribosomal internal transcribed spacer (ITS) region was amplified by the polymerase chain reaction (PCR) using an AccuPower PCR Premix Kit (Bioneer, Seoul, Korea) with the primers, ITS1F [18] and ITS4 [19,20]. If necessary, β-tubulin (TUB) and translation elongation factor-1α (EF1α) region were sequenced using the primers, T10 [21] and Bt2a [22], and EF1-728F [23] and TEF1 rev [24], respectively. DNA sequencing was performed by Macrogen (Seoul, Korea), and the fungi were identified based on a BLAST search (http://blast.ncbi.nlm.nih.gov/Blast.cgi). The DNA sequences of seven selected fungi were deposited in GenBank.

### 2.3. Solid-state culture for prescreening

For primary screening, all the fungi were inoculated on a solid medium containing 39 g of potato dextrose agar (PDA, Bacto, Sparks, MD, USA) in a liter of distilled water (D.W.). They were incubated at room temperature for four weeks because some fungi produce pigments only in a nutritionally deficient state. After cultivation, fungi that colored the medium were selected and subjected to subsequent experiments.

### 2.4. Submerged culture

The selected fungi were precultured on PDA for a week at room temperature (21–23 °C), and three agar plugs (0.6 mm diameter) with actively growing mycelia were used as inocula. They were inoculated 100 ml Erlenmeyer flasks containing 40 ml of 2.4% (w/v) potato dextrose broth (PDB, Bacto, Sparks, MD, USA), and the cultures were maintained for four weeks in a shaking incubator at 150 rpm and 27 °C.

### 2.5. Extraction of fungal pigments and spectrometry

After incubation, 350 μl aliquots of the culture broth were sampled in 2.0 ml e-tubes at seven-day intervals. Four volumes of 95% (v/v) ethanol were added to the samples, which were then mixed vigorously using a vortexer. The tubes were placed in a refrigerator and maintained at 4 °C for an hour to precipitate extracellular polysaccharides. The mixtures were then centrifuged at 4,000 × *g* for 15 min, and the supernatants were filtered through a 0.45 μm syringe filter (Minisart, Sartorius, Göttingen, Germany).

Visible absorption of the filtrates was scanned from 380–800 nm using a Genesys^TM^ 10 UV-Vis spectrophotometer (Thermo Fisher Scientific, Waltham, MA, USA). Units of absorbance (UAs) were calculated by multiplying the maximum absorbance (Abs) by the final dilution factor, thus accurately representing the pigment concentration. The maximum absorbances were found in the ranges from 380–490 nm and from 490–595 nm for yellowish (yellow-green to orange) and reddish (red to purple) pigments, respectively. Pigment production (UA/day) was calculated by dividing the UAs by the number of days spent in culture.

For subsequent assays, the pigment extracts from the fungi with high productivity were dried using a rotary evaporator and then weighed gravimetrically.

### 2.6. Colorimetry

The dried pigment extracts were dissolved in D.W. to concentrations of 1 mg/ml. The CIELAB parameters (L*, a*, b*, C*_ab_, and h) of the samples were measured using a colorimeter (CM-5, Konica Minolta, Tokyo, Japan) based on the protocols of the Commission Internationale de L'Eclairage (CIE).

### 2.7. Antioxidant assay

The antioxidant activity of the pigment extracts from the selected fungi was measured [19]. The dried pigment extracts were dissolved in D.W. to concentrations of 10 mg/ml.

Radical-scavenging activity of 2,2′-azinobis-(3-ethylbenzothiazoline-6-sulfonic acid) (ABTS; Sigma-Aldrich Inc., St. Louis, MO, USA) was analyzed [25]. Briefly, 10 μl of the pigment extract (10 mg/ml) and 990 μl of a prepared ABTS radical solution were added to a cuvette. The absorbance at 734 nm was measured using a UV-vis spectrophotometer after six minutes (Optizen 2120 UV, Mecasys, Daejeon, Korea).

The radical-scavenging activity of 2,2-diphenyl-1-picrylhydrazyl (DPPH; Sigma-Aldrich Inc.) was also measured [26]. Briefly, 22 μl of the pigment extract (10 mg/ml) and 200 μl of a prepared DPPH solution were added to a 96-well plate. The absorbance at 520 nm was measured using a microplate reader after 30 min (Sunrise^TM^, Tecan, Männedorf, Switzerland).

### 2.8. Acute toxicity test

The dried pigment extracts were dissolved in D.W. to concentrations of 10 mg/ml. The acute toxicity test was conducted based on the Organization for Economic Co-operation and Development (OECD) Guideline No. 202 [27]. Briefly, neonates of *Daphnia magna* that were less than 24 h old were used. Five individuals and 10 ml of each sample solution were placed in each well of a 6-well plate and then incubated for 48 h at 20 ± 2 °C without feeding. The immobilization rate of the neonates was used to calculate EC_50_ values by the trimmed Spearman–Karber method. This test was conducted in quadruplicate and validated by a reference test using K_2_Cr_2_O, for which the 48 h EC_50_ was 1.51 mg/L, within the standard sensitivity range (1.23–1.86 mg/L).

### 2.9. Statistical analysis

All the experiments were conducted more than three times, and the mean values are presented. Normality was examined using the Shapiro-Wilk test, and the data were analyzed using one-way ANOVA followed by the post hoc Tukey’s test. Statistical analyses were performed with SAS ver. 9.4 (SAS Institute Inc., Cary, NC, USA), and a p-value below 0.05 indicated a statistically significant difference.

## 3. Results

### 3.1. Primary screening of pigment-producing strains

All the fungal candidates were cultivated on PDA plates for four weeks for prescreening. The cultures were sorted based on the distinct coloration of the medium by macroscopic observation (supplemental Table S1). As a result, 34 fungal strains (19 genera, 33 species) were selected as potential producers of water-soluble pigments and used in subsequent experiments (Table 1). These strains consisted of 8 basidiomycetes (7 genera, 8 species), 22 ascomycetes (9 genera, 21 species), and 4 zygomycetes (3 genera, 4 species). As expected, ascomycetes accounted for the majority (65%) of the pigment-producing fungi.

**Table 1.** The colors of pigments produced by fungal strains in potato dextrose agar.

Fungal species	Strain ID	Observed color
Basidiomycete		
* Crustoderma* sp.	KUC8611	Yellow
* Hyphodontia* sp.	KUC10613	Brown
* Leucogyrophana mollusca*	KUC10761	Yellow
* Phaeoacremonium scolyti*	KUC 9193	Pink
* Phellinus. laevigatus*	KUC10604	Brown
* Phellinus pomaceus*	KUC10654	Brown
* Sanghuangporus baumii*	KUC10644	Brown
* Tyromyces chioneus*	KUC10710	Pink
Ascomycete		
* Aspergillus* sp.	KUC21245	Red
* Chaetomium* sp.	KUC21238	Olive
* Clonostachys. Intermedia*	KUC21274	Yellow
* Grosmannia huntii-like*	KUC2769	Dark Brown
* Helotiales* sp.	KUC21275	Black
* Paecilomyces* sp. 3	KUC1519	Yellow
* Penicillium chermesinum*	KUC21065	Purple
* Penicillium commune*	KUC3029	Yellow
* Penicillium decaturense*	KUC1684	Yellow
* Penicillium miczynskii*	KUC1551	Red
* P. miczynskii*	KUC1721	Red
* Talaromyces funiculosus*	KUC3055	Purple
* Talaromyces pinophilus*	KUC1758	Red
* Talaromyces siamensis*	KUC4096	Red
* Talaromyces* sp.	KUC21114	Yellow
* Trichoderma afroharzianum*	KUC21213	Red
* Trichoderma albolutescens*	KUC21115	Gray
* Trichoderma dorotheae*	KUC21048	Yellow
* Trichoderma longibrachiatum*	KUC21210	Yellow
* Trichoderma pyramidale*	KUC21091	Yellow
* Trichoderma spirale*	KUC21268	Yellow
* Trichoderma* sp. 1	KUC1716	Yellow
Zygomycete		
* Mucor circinelloides*	KUC30035	Yellow
* Mucor mucedo*	KUC1803	Yellow
* Rhizomucor variabilis*	KUC6003	Yellow
* Rhizopus. Arrhizus*	KUC6014	Yellow

### 3.2. Water-soluble pigment production in submerged culture

**Figure 1.**
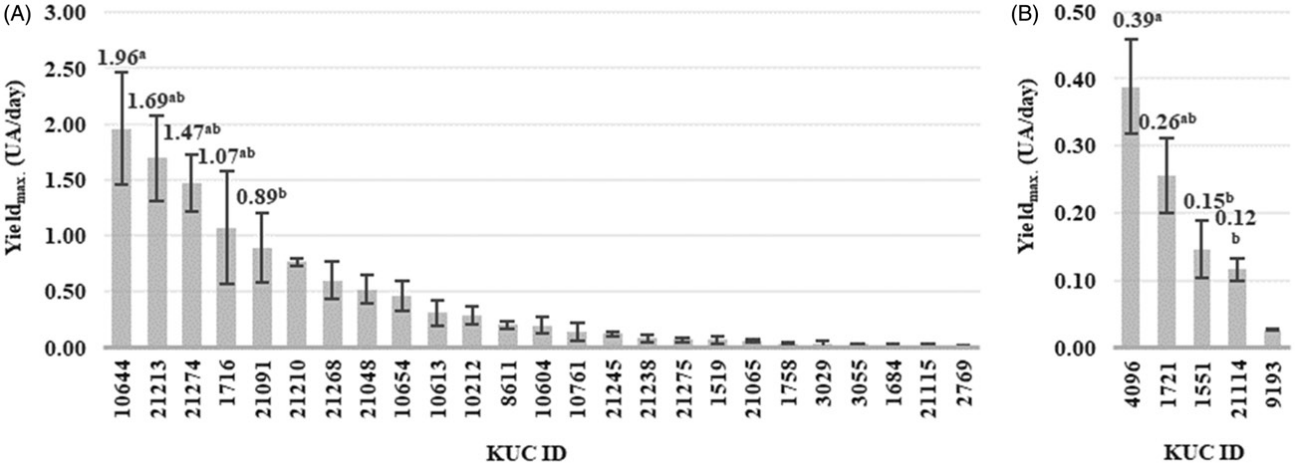
Comparison of pigment yield in submerged culture of selected fungi. (A) from those producing yellowish pigments, (B) from those producing reddish pigments. The error bars indicate the standard deviation. Values with the same letter do not differ significantly (α = 0.05) according to Tukey’s test.

All the selected fungi were cultured in PDB medium for four weeks so that their ability to diffuse pigments into the liquid medium of the submerged culture could be determined. *Sanghuangporus baumii* KUC10644 and *Talaromyces siamensis* KUC4096 exhibited the greatest yields in terms of measured UA/day for yellowish and reddish pigments, respectively (Figure 1).

Among the strains that produced yellowish pigments, *S. baumii* KUC10644 had the highest yield, followed by *Trichoderma afroharzianum* KUC21213, *Clonostachys intermedia* KUC21274, *Trichoderma* sp. 1 KUC1716, and *Trichoderma pyramidale* KUC21091 (Table 2). The pigment productivity of these five strains was higher than that of the 14 least productive strains, with statistical significance. In the case of reddish pigments, *T. siamensis* KUC4096 exhibited the highest yield, followed by *Penicillium miczynskii* KUC1721. The productivity of these two strains was not differentiated with statistical significance.

**Table 2.** Identifying information of the selected fungal strains producing water-soluble pigments in submerged culture.

Fungal species	Strain ID	GenBank accession number	Incubation time_max._^d^
Yellowish-pigment producers			
* Sanghuangporus baumii*	KUC10644	MH168100^a^	14
* Trichoderma afrharzianum*	KUC21213	KX912217^b^	7
* Clonostachys intermedia*	KUC21274	MH168099^a^	14
* Trichoderma* sp. 1	KUC1716	KR820004^b^	7
* Trichoderma pyramidale*	KUC21091	KX912188^b^	7
Reddish-pigment producers			
* Talaromyces siamensis*	KUC4096	MH168102^a^, MH168103^c^	7
* Penicillium miczynskii*	KUC1721	MH168101^a^	28

^a^Internal transcribed spacer (ITS) region.

^b^β-tubulin (TUB) region.

^c^Translation elongation factor-1α (EF1α) region.

^d^The incubation time (day) that exhibited the highest yield.

### 3.3. Colorimetry

We measured the CIELAB parameters to precisely describe the colors of the seven pigment extracts (1 mg/ml) (Table 3). The colors were then expressed by entering the measured parameters into photo editing software (Adobe Photoshop CS6, Adobe Systems Inc., San Jose, CA, USA).

**Table 3.** The CIELAB parameters of the pigment extracts from the selected fungi.

Fungal species	Strain ID	L*	a*	b*	C*	h
*Sanghuangporus baumii*	KUC10644	85.8	−3.3	72.1	72.2	92.6
*Trichoderma afroharzianum*	KUC21213	84.2	0.2	39.4	39.4	89.7
*Clonostachys intermedia*	KUC21274	94.9	−1.7	8.3	8.4	101.6
*Trichoderma* sp. 1	KUC1716	86.5	0.3	44.1	44.1	89.6
*Trichoderma pyramidale*	KUC21091	84.3	−2.1	53.8	53.9	92.2
*Talaromyces siamensis*	KUC4096	88.2	1.8	24.3	24.4	85.7
*Penicillium miczynskii*	KUC1721	73.3	12.6	41.1	42.9	73.0

The parameters were measured at D65 (Daylight 6500K).

### 3.4. Antioxidant activity of the fungal pigment extracts

**Figure 2.**
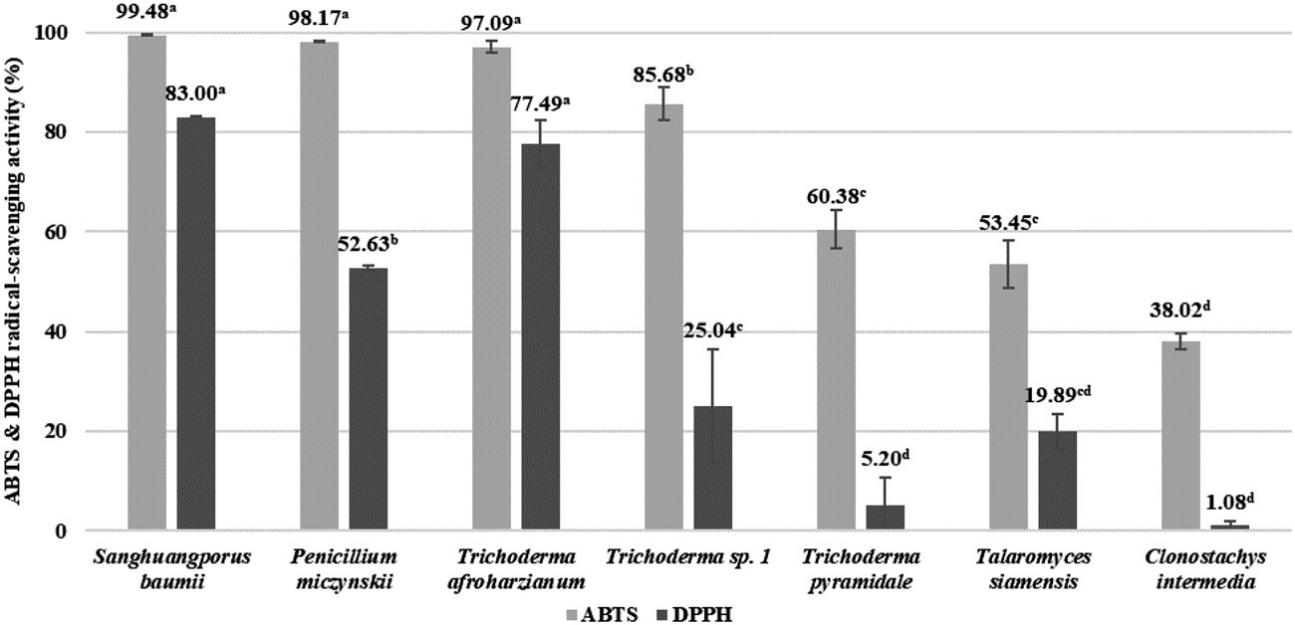
ABTS (left) and DPPH (right) radical-scavenging activity of the pigment extracts. The error bars indicate the standard deviation. Values with the same letter in each group do not differ significantly (α = 0.05) according to Tukey’s test.

To evaluate the functionality of the seven pigment extracts (10 mg/ml), we measured their antioxidant activity (Figure 2). As a result, the highest activities in both the ABTS and DPPH radical-scavenging assays were observed with the pigment extracts from *P. miczynskii* KUC1721, *S. baumii* KUC10644 and *T. afroharzianum* KUC21213, and the *Trichoderma* sp. 1 extract showed high ABTS radical-scavenging activity.

### 3.5. Acute toxicity of the fungal pigment extracts

**Figure 3.**
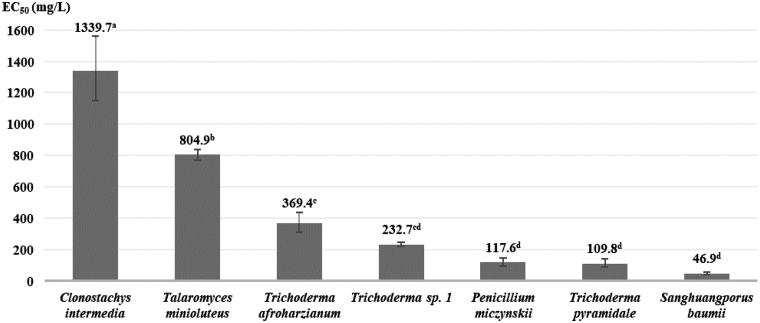
EC_50_ values of the selected pigment extracts against the neonates of *Daphnia magna*. The error bars indicate the 95% confidential intervals. Values with the same letter in each group do not differ significantly (α = 0.05) according to Tukey’s test.

To evaluate the feasibility of the industrial use of the identified fungal pigment extracts, their acute toxicity was measured using neonates of *Daphnia magna*. The environmental impact of disposal of the pigment extracts could be predicted through this analysis, although the human safety cannot be guaranteed. Figure 3 shows the EC_50_ values of the seven selected pigment extracts. A higher EC_50_ value indicates that the sample is less toxic.

## 4. Discussion

### 4.1. Fungal strains producing water-soluble pigments in submerged culture

*S. baumii* KUC10644 and its unknown yellowish pigments have particularly high potential because this organism belongs to the Basidiomycota, which have rarely been studied for their potential to produce fungal pigments from mycelial culture. Pigment and toxin production from the mycelial cultures of genus *Sanghuangporus* has not been previously studied. *C. intermedia* KUC21274 had one of the highest pigment productivities, although there is only one previous report of pigment production from another *Clonostachys* species; unknown yellow pigments were produced from *C. cylindrospora* [28]. In addition, toxins have not been reported to be produced from the genus *Clonostachys*.

The three *Trichoderma* strains (*T. pyramidale* KUC21091, *Trichoderma* sp. 1 KUC1716, and *T. afroharzianum* KUC21213) produced yellowish pigments within seven days, possibly owing to the fast growth of species of the genus *Trichoderma* [29]; they can quickly finish their growth phase and produce secondary metabolites. Since *Trichoderma* species produce yellow anthraquinones, the yellowish pigments from the three strains are likely to be members of this class of molecules [7,30]. However, structural identification should be preceded by a toxicity test of the pigment extracts, given that other *Trichoderma* species produce toxins: T-2 toxin and trichotoxin A40 from *T. viride*, trichodermin from *T. brevicompactum*, trichodermol from *T. polysporum* and *T. reesei*, and gliotoxin (immunosuppressive mycotoxin) from *T. deliquescens*, *T. viride*, and *T. hamatum* [31,32].

Meanwhile, *T. siamensis* KUC4096 produced many reddish pigments within seven days. Considering that several other *Talaromyces* species such as *T. funiculosus*, *T. pinophilus*, and *T. ruber* produce *Monascus*-like azaphilones, the reddish pigments from *T. siamensis* may possibly belong to this class of molecules [33]. *T. siamensis* is thus very likely to also produce citrinin because citrinin production accompanies pigment biosynthesis in *Monascus* species, as mentioned above. In addition, other *Talaromyces* species produce toxins; talarotoxin is produced by *T. bacillosporus*, wortmannin by *T. flavus*, rubratoxins (hepatotoxic mycotoxins) by *T. purpurogenus* and *T. ruber*, kojic acid (potential contact allergen that causes dermatitis) by *T. ruber*, and luteoskyrin (hepatotoxic carcinogen) by *T. rugulosus* [33–38]. Moreover, *T. marneffei* is an opportunistic human pathogen that produces a yellow pigment named secalonic acid D, which is toxic and teratogenic [39,40]. The toxicity of the pigment extracts from *T. siamensis* KUC4096 should therefore be assayed to evaluate the industrial value of this microorganism.

*P. miczynskii* KUC1721 also produced reddish pigments. There is no previous report of reddish pigment produced by this species. Red herqueinone and norherqueinone have been produced in *P. atrovenetum*, and many *Penicillium* species produce *Monascus*-like azaphilones [2,16]. Many mycotoxins are found in *Penicillium* species: plastatin and luteosporin are produced by *P. chermesinum*, and ochratoxin A is produced by *P. aurantiogriseum*, *P. chrysogenum*, *P. nordicum*, and *P. verrucosum* [8,41,42]. *P. miczynskii* itself has also been reported to produce mycotoxins such as citrinin, citreoviridin, citreomontanin, and penicillic acid [43]. In contrast, ethyl acetate extracts of some *Penicillium* species had no toxic effects on *Artemia salina* larvae [14]. This result implies that *P. miczynskii* strain KUC1721 may not produce toxic compounds or toxin production may be low, but the toxicity of this pigment extract must be thoroughly tested because the extraction solvent used was different.

### 4.2. Pigment accumulation and color development

The accumulation of pigments caused the culture media with yellowish pigments to become reddish, but the medium inoculated with *C. intermedia* KUC21274 maintained its yellowish color. The yellowish pigments may have been produced vigorously for two weeks and then decomposed or derivatized so that they did not accumulate, even if the incubation was prolonged; or the pH of the medium might have changed. This hypothesis is supported by the UA of the pigment extract from *C. intermedia* KUC21274 decreasing after the second week of incubation (data not shown). The expressed color of the pigment extract from *C. intermedia* KUC21274 was very light due to the reasons described above.

### 4.3. Fungal pigment extracts with high antioxidative ability

In the case of *S. baumii*, several reports describe its physiological activity, including antioxidant, anti-inflammatory, and hypoglycemic effects, and the fruiting body of this fungus is well known as a medicinal mushroom. However, none of these reports examined the extracellular pigment extracts from the culture filtrate, but rather the organism’s fruiting bodies, mycelial extracts, and polysaccharides [44–48]. For example, the high antioxidant activity of a water-soluble extract of *S. baumii* has been reported, but the experimental sample was a mycelial extract that had been prewashed several times [48]. The high antioxidant activity was therefore speculated to have resulted from the pigment itself or another non-polysaccharide compound. High antioxidant activity has not been reported for *P. miczynskii*, *Trichoderma* sp. 1, or *T. afroharzianum* extracts. Whether these antioxidants are pigments themselves or other secondary metabolites must therefore be confirmed, and other physiological characteristics such as anti-fungal and antibacterial activity must be tested. These results imply that the pigment extracts from these four fungi have potential as functional colorants applicable to the food and cosmetic industries.

### 4.4. Environmental toxicity of fungal pigment extracts

EC_50_ values are generally interpreted as presented in Table 4 [49]. The table shows that the pigment extract from *S. baumii* was moderately toxic, while the other six extracts were relatively non-toxic. The pigment extracts from *C. intermedia* and *T. siamensis* exhibited significantly lower toxicity than the others did. This result showed that the use of these six pigment extracts is industrially feasible in terms of the cost reduction of wastewater treatment. It is suggested that their toxicity to humans should be tested to make them available for applications in the cosmetic and food industries without safety problems. On the other hand, the pigment extract from *S. baumii* appears to have limited application in industry due to its toxicity. The toxic substance from *S. baumii* should be identified, as no toxicity report on this fungal genus exists.

**Table 4.** The general toxicity rating corresponding to EC_50_ value.

EC_50_ (mg/L)	Toxicity rating
>100	Relatively non-toxic
10–100	Moderately toxic
1–10	Very toxic
<1	Extremely toxic

### 4.5. Fungal strains producing safe functional water-soluble pigments

Five fungal species (*Sanghuangporus baumii*, *Clonostachys intermedia*, *Trichoderma pyramidale*, *Trichoderma* sp. 1, and *Trichoderma afroharzianum*) produced intense yellowish pigments, and two species (*Talaromyces siamensis* and *Penicillium miczynskii*) produced intense reddish pigments. The pigment extracts from *P. miczynskii*, *S. baumii*, *Trichoderma* sp. 1, and *T. afroharzianum* exhibited high antioxidant activity, suggesting the potential to serve as functional colorants for the food and cosmetic industries. Among them, all the extracts except for the one from *S. baumii* were relatively non-toxic in the acute toxicity test. In conclusion, *P. miczynskii* KUC1721, *Trichoderma* sp. 1 KUC1716, and *T. afroharzianum* KUC21213 were the best fungal strains identified for the industrial production of safe functional water-soluble pigments. This is the first report that evaluated the productivity, functionality, and environmental toxicity of water-soluble pigments from the selected fungal species. To further ensure their industrial value, their human toxicity must be studied, and their pigment yields must be maximized by optimizing culture and extraction conditions.

## Supplementary Material

Supplemental Material
