# Supplementary material for: Investigation of Filamentous Fungi Producing Safe, Functional Water-Soluble Pigments
Source: Mycobiology. 2018 Sep 27;46(3):269–77. doi: 10.1080/12298093.2018.1513114 (PMC6171424; doi:10.1080/12298093.2018.1513114)
Supplement: Supplemental Material [file TMYB_A_1513114_SM7498.doc]

**SUPPLEMENTAL TABLE**

**Table S1** Potential pigment producer candidate list and the reasons for selection

| **Fungal species** | **KUC ID** | **Color^a^** | **Reported pigments** | **References** |
| --- | --- | --- | --- | --- |
| **Basidiomycete** |  |  |  |  |
| ***Crustoderma*** |  |  |  |  |
| *Crustoderma* sp. | 8611 | Yellow |  |  |
| ***Gloeophyllum*** |  |  |  |  |
| *G. abietinum* | 8053 | Yellow |  |  |
| ***Hyphodontia*** |  |  |  |  |
| *H. radula* | 10306 | Brown |  |  |
| *H. tropica* | 10071 |  |
| *Hyphodontia* sp. | 10613 |  |
| ***Leucogyrophana*** |  |  |  |  |
| *L. mollusca* | 10761 | Yellow |  |  |
| ***Panellus*** |  |  | *P. serotinus*: 3-methylriboflavin |  |
| *P. stipticus* | 8834 |  |  |  |
| ***Phaeoacremonium*** |  |  |  |  |
| *P. scolyti* | 9193 | Pink |  |  |
| ***Phanerochaete*** |  |  |  |  |
| *P. sordida* | 10704 | Yellow |  |  |
| ***Phellinus*** |  |  | Fruiting body of *P. igniarius*: phenolic pigments  Fruiting body of *P. weirii*: melanin-like pigments |  |
| *P. laevigatus* | 10604 |  |  |  |
| *P. pomaceus* | 10654 | Brown |  |  |
| ***Pycnoporus*** |  |  | *P. cinnabarinus, P. coccineus*: phenoxazinones |  |
| *Pycnoporus* sp. | 9076 |  |  |  |
| ***Sanghuangporus*** |  |  |  |  |
| *S. baumii* | 10644 | Brown |  |  |
| ***Tyromyces*** |  |  |  |  |
| *T. chioneus* | 10710 | Pink |  |  |
| **Ascomycete** |  |  |  |  |
| ***Alternaria*** |  |  | Anthraquinones (yellow, red)  *A. porri*: altersolanol A (yellow)-C, alterporriol B  *A. solani*, *A. tomatophila*: altersolanol A (yellow) |  |
| *A. alternata* | 21222 |  |  |  |
| *A. tenuissima* | 5143 |  |  |  |
| *Alternaria* sp. 1 | 5029 |  |  |  |
| *Alternaria* sp. 2 | 21049 |  |  |  |
| *Alternaria* sp. 3 | 21060 |  |  |  |
| ***Aspergillus*** |  |  | *A. melleus*, *A. sulphureus*, *A. westerdijkiae*: rubrosulphin (red, toxic), viomellein (reddish-brown, toxic), viopurpurin (purple, toxic), xanthomegnin (orange, toxic)  *A. terreus*: citrinin (yellow, toxic) |  |
| *A. flavipes* | 5033 |  |  |  |
| *A. japonicas* | 5035 |  |  |  |
| *A. tubingensis* | 5037 |  |  |  |
| *A. wentii* | 5203 |  |  |  |
| *Aspergillus* sp. | 21245 |  |  |  |
| ***Beauveria*** |  |  | Oosporein (red), tenellin, bassianin (yellow) |  |
| *Beauveria* sp. | 21117 |  |  |  |
| ***Ceratocystiopsis*** |  |  | *Ophiostoma*-like fungi |  |
| *C. minuta-like* | 2619 |  |  |  |
| *C. pilifera* | 2766 |  |  |  |
| *C. pluriannulata-like* | 2795 |  |  |  |
| ***Chaetomium*** |  |  | *C. cochliodes*: cochliodinol (purple)  *C. trilaterale*, *C. aureum*: oosporein (red) |  |
| *C. murorum* | 21225 |  |  |  |
| *Chaetomium* sp. | 21238 |  |  |  |
| ***Clonostachys*** |  |  | *C. cylindrospora*: unknown yellow pigments |  |
| *C. intermedia* | 21274 |  |  |  |
| ***Graphilbum*** |  |  |  |  |
| *G. fragrans* | 2080 |  | Unknown yellow pigment |  |
| ***Grosmannia*** |  |  | *G. wageneri*: hydroxyvertixanthone (yellow), 1,3,6,8-tetrahydroxyanthraquinone (orange-red) |  |
| *G. huntii-like* | 2769 |  |  |  |
| ***Helotiales*** |  |  |  |  |
| *Helotiales* sp. | 21275 | Black |  |  |
| ***Hyalorhinocladiella*** |  |  | *Ophiostoma*-like fungi |  |
| *Hyalorhinocladiella* sp. | 2527 |  |  |
| ***Isaria*** |  |  | *I. farinosa*: anthraquinone (red) |  |
| *I. javanica* | 21075 |  |  |  |
| ***Leptographium*** |  |  | *Ophiostoma*-like fungi |  |
| *L. koreanum* | 2610 |  |  |  |
| *L. piceaperdum* | 2929 |  |  |  |
| ***Neurospora*** |  |  | *N. crassa*: carotenoid |  |
| *N. intermedia* | 5175 |  |  |  |
| *Neurospora* sp. | 5086 |  |  |  |
| ***Paecilomyces*** |  |  | *P. sinclairii*: unknown red pigment |  |
| *Paecilomyces* sp. 1 | 5262 |  |  |  |
| *Paecilomyces* sp. 2 | 1518 |  |  |  |
| *Paecilomyces* sp. 3 | 1519 | Yellow |  |  |
| ***Penicillifer*** |  |  | *P. pulcher*: unknown brown pigments |  |
| *P. diparietisporus* | 21278 |  |  |  |
| ***Penicillium*** |  |  | *Monascus*-like azaphilones  *P. atrovenetum*: herqueinone, norherqueinone (red), unknown bluish green pigments  *P. herquei*: atrovenetin (yellow)  *P. persicinum*: unknown reddish pink pigments  *P. fagi*: unknown greenish blue pigments  *P. verrucosum*: citrinin (yellow, toxic) |  |
| *P. brasilianum* | 5057 |  |  |  |
| *P. chermesinum* | 21120 |  |  |  |
| *P. chermesinum* | 21065 |  | Plastatin (orange red), luteosporin (black) |  |
| *P. commune* | 3029 |  |  |  |
| *P. decaturense* | 1684 |  |  |  |
| *P. dierckxii* | 5150 |  |  |  |
| *P. glabrum* | 5159 |  |  |  |
| *P. glaucoalbidum* | 21175 |  |  |  |
| *P. implicatum* | 1619-2 |  |  |  |
| *P. meleagrinum var. viridiflavum* | 1730 |  |  |  |
| *P. miczynskii* | 1551 |  |  |  |
| *P. miczynskii* | 1721 |  |  |  |
| *P. multicolor* | 1626 |  |  |  |
| *P. ochrochloron* | 1480 |  |  |  |
| *P. purpurascens* | 3038 |  |  |  |
| *P. raistrickii* | 1729 |  |  |  |
| *P. simplicissimum* | 5153 |  |  |  |
| *P. simplicissimum* | 5268 |  |  |  |
| *P. solitum* | 3019 |  |  |  |
| *P. sumatraense* | 1620 |  |  |  |
| *Penicillium* sp. 1 | 1473 |  |  |  |
| *Penicillium* sp. 2 | 1651 |  |  |  |
| *Penicillium* sp. 3 | 1759 |  |  |  |
| *Penicillium* sp. 4 | 1795-3 |  |  |  |
| ***Pesotum*** |  |  | Anamorph of *Ophiostoma* |  |
| *P. piceae* | 2928 |  |  |  |
| *P. piceae* | 20005 |  |  |  |
| *P. piceae* | 20009 |  |  |  |
| ***Purpureocillium*** |  |  |  |  |
| *P. lilacinum* | 5007 | Yellow |  |  |
| ***Sporothrix*** |  |  | *S. schenckii*: melanin-like pigments  *S. brunneoviolacea*: unknown violet-brown pigments |  |
| *Sporothrix* sp. | 2217 |  |  |  |
| ***Talaromyces*** |  |  | *T. aculeatus*, *T. purpureogenus*, *T. amestolkiae*, *T. stollii*: *Monascus*-like azaphilones |  |
| *T. diversus* | 1728 |  |  |  |
| *T. funiculosus* | 3055 |  | *Monascus*-like azaphilones |  |
| *T. pinophilus* | 1758 |  | *Monascus*-like azaphilones |  |
| *T. pinophilus* | 21276 |  |
| *T. radicus* | 5059 |  |  |  |
| *T. siamensis* | 4096 |  |  |  |
| *T. variabilis* | 1757 |  |  |  |
| *T. verruculosus* | 1794 |  |  |  |
| *Talaromyces* sp. | 21114 |  |  |  |
| ***Trichoderma*** |  |  | *T. polysporum*: anthraquinones (yellow)  Unknown red pigments |  |
| *T. afroharzianum* | 21213 |  |  |  |
| *T. albolutescens* | 21115 |  |  |  |
| *T. asperelloides* | 21191 |  |  |  |
| *T. asperellum* | 21206 |  |  |  |
| *T. atroviride* | 21002 |  |  |  |
| *T. aureoviride* | 21005 |  |  |  |
| *T. cerinum* | 21111 |  |  |  |
| *T. citrinoviride* | 21022 |  |  |  |
| *T. dorotheae* | 21048 |  |  |  |
| *T. harzianum* | 21174 |  |  |  |
| *T. koningii* | 5285 |  |  |  |
| *T. longibrachiatum* | 5222 |  |  |  |
| *T. longibrachiatum* | 21210 |  |  |  |
| *T. orientale* | 21216 |  |  |  |
| *T. pyramidale* | 21091 |  |  |  |
| *T. spirale* | 21268 |  |  |  |
| *Trichoderma* sp. 1 | 1716 |  |  |  |
| *Trichoderma* sp. 2 | 21142 |  |  |  |
| **Zygomycete** |  |  |  |  |
| ***Gongronella*** |  |  | *Mucor*-like fungi |  |
| *G. butleri* | 6007 |  |  |  |
| ***Mucor*** |  |  | β-carotene (carotenoid, yellow) |  |
| *M. circinelloides* | 30035 |  |  |  |
| *M. fragilis* | 30033 |  |  |  |
| *M. hiemalis* | 30039 |  |  |  |
| *M. mucedo* | 1803 |  |  |  |
| *M. plumbeus* | 5087 |  |  |  |
| *M. racemosus* | 6001 |  |  |  |
| ***Mucoromycotina*** |  |  | *Mucor*-like fungi |  |
| *Mucoromycotina* sp. | 6002 |  |  |  |
| ***Rhizomucor*** |  |  | *Mucor*-like fungi |  |
| *R. variabilis* | 6003 |  |  |  |
| ***Rhizopus*** |  |  |  |  |
| *R. arrhizus* | 6014 | Yellow |  |  |
| *R. stolonifera* | 1413 |  |  |  |
| *Rhizopus* sp. 1 | 1705 |  |  |  |
| *Rhizopus* sp. 2 | 1731 |  |  |  |

^a^ The color that appeared at random observation of fungal cultures from the Korea University Culture (KUC) collection.

**SUPPLEMENTAL LITERATURE CITED**

1. Steglidi W, Zechlin L. Pigments of Fungi, XXXII. 3-Methylriboflavine from *Panellus serotinus* (Agaricales). Zeitschrift für Naturforschung C 1977;32:520-2.

2. Kirk TK, Lorenz LF, Larsen MJ. Partial characterization of a phenolic pigment from sporocarps of *Phellinus igniarius*. Phytochemistry 1975;14:281-4.

3. Li CY. Melanin-like pigment in zone lines of *Phellinus weirii*-colonized wood. Mycologia 1983;75:562-6.

4. Gross B, Yonnet G, Picque D, Brunerie P, Corrieu G, Asther M. Production of methylanthranilate by the basidiomycete Pycnoporus cinnabarinus (Karst.). Appl Microbiol Biotechnol 1990;34:387-91.

5. Mapari SAS, Meyer AS, Thrane U, Frisvad JC. Identification of potentially safe promising fungal cell factories for the production of polyketide natural food colorants using chemotaxonomic rationale. Microbial Cell Factories 2009;8:24.

6. Suemitsu R, Nakamura A, Isono F, Sano T. Isolation and Identification of Dactylariol from the Culture Liquid of *Alternaria porri* (Ellis) Ciferri. Agric Biol Chem 1982;46:1693-4.

7. Suemitsu R, Sano T, Yamamoto M, Arimoto Y, Morimatsu F, Nabeshima T. Structural elucidation of alterporriol B, a novel metabolic pigment produced by *Alternaria porri* (Ellis) ciferri. Agric Biol Chem 1984;48:2611-3.

8. Conde FP, Orlandi R, Canevari S, Mezzanzanica D, Ripamonti M, Muñoz SM, Jorge P, Colnaghi MI. The *Aspergillus* toxin restrictocin is a suitable cytotoxic agent for generation of immunoconjugates with monoclonal antibodies directed against human carcinoma cells. The FEBS Journal 1989;178:795-802.

9. Mapari SAS, Thrane U, Meyer AS. Fungal polyketide azaphilone pigments as future natural food colorants? Trends Biotechnol 2010;28:300-7.

10. Basyouni SHE, Brewer D, Vining LC. Pigments of the genus *Beauveria*. Can J Bot 1968;46:441-8.

11. Wat C-K, Mcinnes AG, Smith DG, Wright JLC, Vining LC. The yellow pigments of *Beauveria* species. Structures of tenellin and bassianin. Canadian Journal of Chemistry 1977;55:4090-8.

12. Brewer D, Jerram WA, Taylor A. The production of cochliodinol and a related metabolite by *Chaetomium* species. Can J Microbiol 1968;14:861-6.

13. Eyal J, Mabud MA, Fischbein KL, Walter JF, Osborne LS, Landa Z. Assessment of *Beauveria bassiana* Nov. EO-1 strain, which produces a red pigment for microbial control. Appl Biochem Biotechnol 1994;44:65-80.

14. Hosoya T, Matsuoka T, Serizawa N, Furuya K. Two morphological groups derived from *Clonostachys cylindrospora* and their relationship to trans-4-hydroxy-(L)-proline productivity. Mycoscience 1995;36:193-7.

15. Jacobs K, Seifert KA. Fungi Canadenses No. 347 *Pesotum Fragrans*. Can J Plant Pathol 2004;26:79-80.

16. Ayer WA, Browne LM, Lin G. Metabolites of *Leptographium* *wageneri*, the Causative Agent of Black Stain Root Disease of Conifers. J Nat Prod 1989;52:119-29.

17. Via BK, Eckhardt LG, So C-L, Shupe TF, Groom LH, Stine M. The response of visible/near infrared absorbance to wood-staining fungi. Wood and fiber science 2007;38:717-26.

18. Velmurugan P, Lee YH, Nanthakumar K, Kamala‐Kannan S, Dufossé L, Mapari SAS, Oh BT. Water‐soluble red pigments from *Isaria farinosa* and structural characterization of the main colored component. J Basic Microbiol 2010;50:581-90.

19. Ogbonna CN. Production of food colourants by filamentous fungi. Afr J Microbiol Res 2016;10:960-71.

20. Cho YJ, Park JP, Hwang HJ, Kim SW, Choi JW, Yun JW. Production of red pigment by submerged culture of *Paecilomyces sinclairii*. Lett Appl Microbiol 2002;35:195-202.

21. Samuels GJ. *Nectria* and *Penicillifer*. Mycologia 1989;81:347-55.

22. Bragulat MR, Martínez E, Castellá G, Cabañes FJ. Ochratoxin A and citrinin producing species of the genus *Penicillium* from feedstuffs. Int J Food Microbiol 2008;126:43-8.

23. Mapari SAS, Nielsen KF, Larsen TO, Frisvad JC, Meyer AS, Thrane U. Exploring fungal biodiversity for the production of water-soluble pigments as potential natural food colorants. Curr Opin Biotechnol 2005;16:231-8.

24. Singh PD, Johnson JH, Aklonis CA, Bush K, Fisher SM, O'Sullivan J. Two new inhibitors of phospholipase A2 produced by *Penicillium chermesinum*. Taxonomy, fermentation, isolation, structure determination and biological properties. The Journal of antibiotics 1985;38:706-12.

25. Madrid H, Gené J, Cano J, Silvera C, Guarro J. *Sporothrix brunneoviolacea* and *Sporothrix dimorphospora*, two new members of the *Ophiostoma stenoceras*-*Sporothrix schenckii* complex. Mycologia 2010;102:1193-203.

26. Morris-Jones R, Youngchim S, Gomez BL, Aisen P, Hay RJ, Nosanchuk JD, Casadevall A, Hamilton AJ. Synthesis of melanin-like pigments by *Sporothrix schenckii* in vitro and during mammalian infection. Infect Immun 2003;71:4026-33.

27. Frisvad JC, Yilmaz N, Thrane U, Rasmussen KB, Houbraken J, Samson RA. *Talaromyces atroroseus*, a new species efficiently producing industrially relevant red pigments. PLoS One 2013;8:e84102.

28. Donnelly DMX, Sheridan MH. Anthraquinones from *Trichoderma polysporum*. Phytochemistry 1986;25:2303-4.

29. Dufosse L, Fouillaud M, Caro Y, Mapari SAS, Sutthiwong N. Filamentous fungi are large-scale producers of pigments and colorants for the food industry. Curr Opin Biotechnol 2014;26:56-61.
